# Selective recording of physiologically evoked neural activity in a mixed autonomic nerve using a minimally invasive array

**DOI:** 10.1063/5.0164951

**Published:** 2023-11-03

**Authors:** Sophie C. Payne, Peregrine B. Osborne, Alex Thompson, Calvin D. Eiber, Janet R. Keast, James B. Fallon

**Affiliations:** 1 Bionics Institute, Victoria 3002, Australia; 2 Medical Bionics Department, University of Melbourne, Victoria 3010, Australia; 3 Department of Anatomy and Physiology, University of Melbourne, Victoria 3010, Australia; 4 Department of Otolaryngology, University of Melbourne, Victoria 3010, Australia

## Abstract

Real-time closed-loop control of neuromodulation devices requires long-term monitoring of neural activity in the peripheral nervous system. Although many signal extraction methods exist, few are both clinically viable and designed for extracting small signals from fragile peripheral visceral nerves. Here, we report that our minimally invasive recording and analysis technology extracts low to negative signal to noise ratio (SNR) neural activity from a visceral nerve with a high degree of specificity for fiber type and class. Complex activity was recorded from the rat pelvic nerve that was physiologically evoked during controlled bladder filling and voiding, in an extensively characterized *in vivo* model that provided an excellent test bed to validate our technology. Urethane-anesthetized male rats (n = 12) were implanted with a four-electrode planar array and the bladder instrumented for continuous-flow cystometry, which measures urodynamic function by recording bladder pressure changes during constant infusion of saline. We demonstrated that differential bipolar recordings and cross-correlation analyses extracts afferent and efferent activity, and discriminated between subpopulations of fibers based on conduction velocity. Integrated Aδ afferent fiber activity correlated with bladder pressure during voiding (r^2^: 0.66 ± 0.06) and was not affected by activating nociceptive afferents with intravesical capsaicin (r^2^: 0.59 ± 0.14, *P* = 0.54, and n = 3). Collectively, these results demonstrate our minimally invasive recording and analysis technology is selective in extracting mixed neural activity with low/negative SNR. Furthermore, integrated afferent activity reliably correlates with bladder pressure and is a promising first step in developing closed-loop technology for bladder control.

## INTRODUCTION

Peripheral nerves projecting to visceral organs are a major therapeutic target for closed-loop devices (Cracchiolo *et al.*, 2021). These nerves are a major brain–body interface, transmitting both sensory (afferent) signals that monitor the physiological and pathophysiological state of the body, and the motor (efferent) signals that directly control visceral organ function during state-dependent feedback (homeostasis) or feedforward (allostasis) brain regulation (Schulkin and Sterling, 2019).

Many peripheral nerve interface arrays that record neural activity from large myelinated peripheral nerves regulating somatic structures—such as TIME (transverse intrafascicular multichannel electrode), SPINE (slowly penetrating interfascicular nerve electrode), and USEA (Utah slanted electrode array)—are designed to penetrate the epineurium to improve selectivity of recording (Fallon and Carter, 2016; Larson and Meng, 2020; and Naufel *et al.*, 2020). However, this increases the risk of compromising the integrity or long-term function of visceral (autonomic) nerves, which are typically smaller and more fragile than somatic nerves. Extraneural electrodes (planar or cuff designs, e.g., the FINE array) are less invasive and chronically viable, but a trade-off is a reduced signal to noise ratio (SNR) (Tyler and Durand, 2002; Paggi *et al.*, 2021). Regardless of the electrode technology used, recording from a single location in a nerve provides no information on the type (afferent vs efferent) or class nerve activity recorded. The velocity selective recording (VSR) approach often extracts large neural signals with a positive SNR acquired through electrically evoking the nerve (i.e., ECAPS). The VSR approach has been demonstrated to be able to differentiate between both fiber type and class by analyzing electrically evoked activity from a 14 multi-electrode array in the ulnar (somatic) nerve in pigs (Andreis *et al.*, 2021; Metcalfe *et al.*, 2021). Although VSR has been demonstrated to extract naturally occurring neural activity during respiration from the large, robust vagus nerve of pigs, this required the use of a ten-multi channel electrode (Metcalfe *et al.*, 2018). Therefore, the ability to extract low levels of neural activity with a low or negative SNR, using a four-channel electrode array appropriate for a small, fragile visceral nerve represents an unmet challenge in the field and is addressed in this study.

Previously, we have developed a minimally invasive, extra-epineural neural interface and have recently reported that it can record electrically evoked neural activity (Table I) from two visceral nerves—the vagus (Payne *et al.*, 2018, 2019, 2022) and pelvic nerve (Payne *et al.*, 2020). Furthermore, we have shown our four-channel electrode “Longitudinal Interface Nerve Electrode” (LINE) device can be safely implanted onto a fragile visceral nerve for periods in excess of 8 weeks (Payne *et al.*, 2020). This array, therefore, provides another alternative for interfacing with small peripheral visceral nerves. However, the challenge of the extraction of low levels of neural activity with a low or negative SNR remains unmet. The broad aim of this study was, therefore, to assess the ability of LINE, as an exemplar array, to extract spontaneous or physiologically evoked neural activity with low/negative SNR from outside the epineurium that is usually too small to be detected against background noise.

**TABLE I.** Summary analysis of electrically evoked waveforms recorded by LINE devices implanted on rat pelvic nerve. Threshold, latency, and conduction velocity data show mean ± SEM.

	Electrode configuration					
	S12R34			S34R12		
Neural population^a^	P1	P2	P3	P1	P2	P3
Threshold (*μ*A)	528 ± 96.5	695 ± 115	722 ± 234.8	356 ± 58.3	696 ± 95.3^b^	670 ± 124.8
Charge density (nC/mm²)	147 ± 26.8	193 ± 31.9	201 ± 65.2	99 ± 16.2	193 ± 26.5^b^	186 ± 34.7
Recording efficiency (%)^c^	92	100	42	67	100	58
Latency (ms)	1.4 ± 0.11	3.0 ± 0.17	5.3 ± 0.22	1.3 ± 0.14	3.0 ± 0.20	5.3 ± 0.22
Conduction velocity (m/s)^d^	2.6 ± 0.24	1.2 ± 0.07	0.64 ± 0.07	2.6 ± 0.24	1.1 ± 0.07	0.6 ± 0.03

^a^ Population peak of waveform evoked by stimulating E1 and E2 (closest to the bladder) and recording from E3 and E4 (S12R34), and vice versa (S34R12).

^b^P2 vs P1, *P* < 0.05, n = 12 rats in total tested, mixed-effect model and *post hoc* Sidak's test.

^c^ Frequency (% out of a total of 12 rats) of a response.

^d^ Conduction velocity estimated using center-to-center distance of 3.25 mm between electrode pairs E1–E2 and E3–E4.

**FIG. 1.**
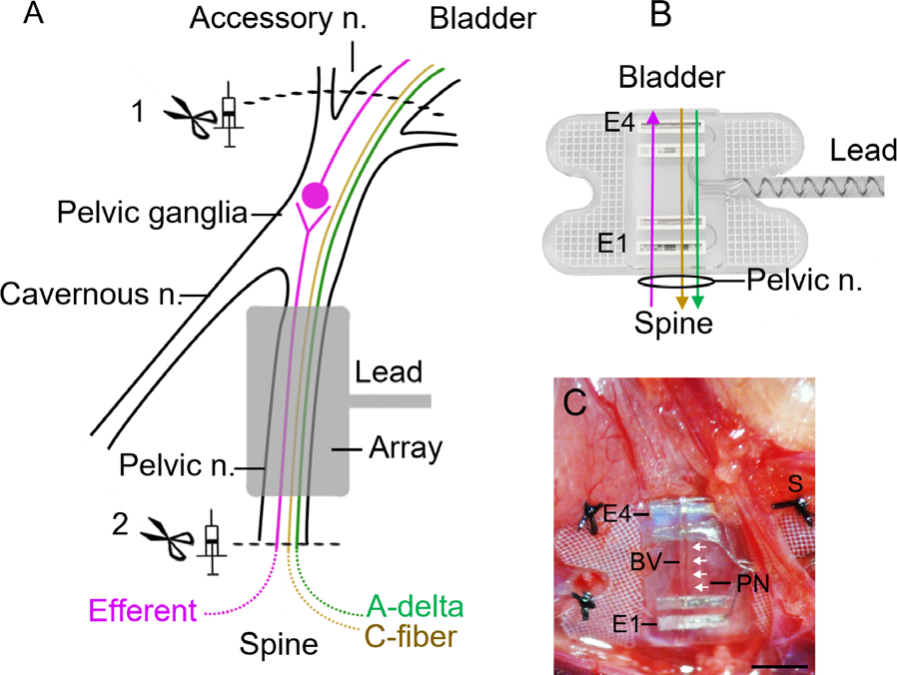
Pelvic nerve anatomy and LINE device. (a) Schematic shows the pelvic nerve and the location of the electrode array. The pelvic nerve contains sensory (afferent, green and brown lines) axons and parasympathetic preganglionic (efferent, pink line) axons that project to and from the bladder, respectively, via the accessory nerve. Scissors and syringe icons indicate the location in which a mechanical injury (crush/lesion) of the accessory or pelvic nerves or pharmacological drugs (TTX, lidocaine) were used to selectively manipulate afferent (1) or efferent (2) activity, respectively. (b) A rendered drawing of the Longitudinal Interface Nerve Electrode (LINE) array used to record afferent (indicated by green/brown lines) and efferent (indicated by a pink line) activity. (c) An *in vivo* image showing the placement of the array's electrodes (E1–E4) perpendicular to the pelvic nerve (PN, indicated by white arrow heads) and secured using sutures (S) and a blood vessel (BV). Scale bar represents: 2 mm.

**FIG. 2.**
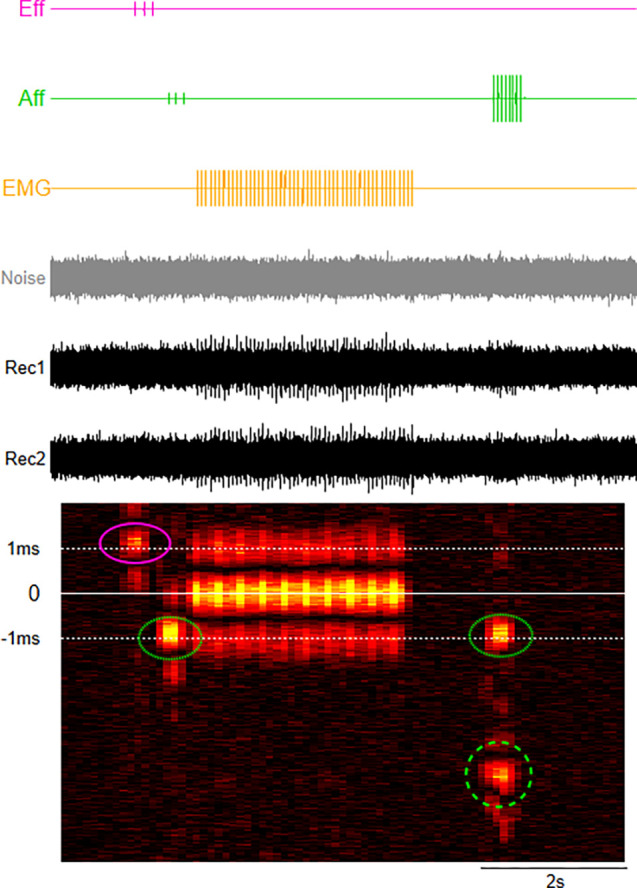
A synthetic model of our cross-correlation signal processing method. Synthetic recordings (Rec 1, Rec 2) were generated by combing stereotypical neural signals from efferent (Eff: pink trace), fast- and slow-afferent (Aff: green trace), and electromyographic (EMG: yellow trace) activity and Gaussian noise (gray trace**)**. The output of the cross correlation between two pairs of recordings (Rec 1, Rec 2) is a heat map, with warmer colors representing more neural activity. The time lag of the peak correlation indicates type of neural activity (fast vs slow), while the sign of the lag (positive or negative) indicates class (sensory vs motor). Here, our model shows efferent activity (Eff, pink circle, ∼1 ms) and two populations of afferent (Aff, fast dotted green circle, ∼1 ms; slow dashed green circle, ∼4 ms) activity, as well as electromyographic (EMG) activity (lag 0 ms).

To address this limitation, this study investigated if a novel signal processing method (see details below) that runs cross-correlation analyses between two pairs of recordings (Fallon, 2019) can extract physiologically evoked neural activity from signals recorded from a mixed autonomic nerve. To do this, we implanted our LINE devices on the pelvic nerve in adult male rats and physiologically evoked neural activity by continuous flow cystometry as reported previously (Payne *et al.*, 2020). The pelvic nerve of rats is well characterized as it is the primary visceral nerve used by lumbosacral afferents (sensory) and parasympathetic autonomic preganglionic efferents (visceral motor) to project to the bladder, distal colon, and reproductive organs (Nadelhaft and Booth, 1984; Keast, 2006) [Fig. 1(a)]. Light and electronic microscopy analysis shows more than 80% of pelvic nerve axons are unmyelinated—which comprise slightly less than half of the sensory fibers; around 80% of the preganglionic fibers, and 100% of a small population of vascular autonomic postganglionic fibers originating from paravertebral sympathetic chain neurons (Purinton *et al.*, 1976; Hulsebosch and Coggeshall, 1982). Electrophysiological recordings also provide functional evidence of C-fibers (0.15–1.3 m/s) and Aδ-fibers (1.3–21 m/s) present in rat pelvic nerve (Purinton *et al.*, 1976; Vera and Nadelhaft, 1990; Sengupta and Gebhart, 1994; and Shea *et al.*, 2000). Complex, sequential pelvic nerve activity can be physiologically evoked during controlled filling and voiding induced by infusing the bladder with saline (i.e., continuous-flow cytometry) (Andersson *et al.*, 2011; Fraser *et al.*, 2020). During evoked voiding in anesthetized rats, afferent activity is minimal during filling, but then markedly increases as pressure rises preceding and during the voiding contraction and effectively ceases during the post void relaxation phase (Le Feber *et al.*, 1997, 2004; Zvara *et al.*, 2010; and Choudhary *et al.*, 2015). When the autonomic motor pathway has been studied by recording post-ganglionic efferent fibers in bladder nerves their activity also correlates with an increase in pressure during the void contraction but ceases during the post void relaxation phase (Le Feber *et al.*, 1997; Choudhary *et al.*, 2015). As such, the first specific aims of this study were to determine if the LINE device and cross-correlation analysis (Fig. 2) could selectively discriminate (1) afferent and efferent neural activity and (2) different fiber classes (Aδ or C) when activated physiologically during cystometry testing. The selectivity of the recording and extraction was validated using various pharmacological and physical interventions to preferentially increase, decrease, or silence various subclasses of neural populations.

A major challenge for developing a robust closed-loop system for bladder control is to determine reliable, objective, real-time sensory feedback to monitor and predict bladder fullness (Cracchiolo *et al.*, 2021). Recent studies in cat show bladder afferent neurons in the sacral dorsal root ganglia (DRG), recorded using penetrating intraneural electrodes, strongly correlate with bladder pressure during isovolumetric testing (Ross *et al.*, 2016; Ross *et al.*, 2018; and Sperry *et al.*, 2021). Recordings from sacral DRG neurons were used to feed a Kalman filter-based non-linear model to predict bladder pressure with a high correlation in an active bladder model leading to closed-loop stimulation of pudendal nerves and increasing bladder capacity (Ouyang *et al.*, 2019). Although informative, the use of intrafascicular penetrating electrodes to record afferent activity is highly invasive and would present a challenge to be translated into the clinic. As such, the second aim of the present study was to correlate extracted afferent activity, using our clinically viable LINE array and cross-correlation analysis, with physiologically evoked bladder pressure during cystometry testing.

## RESULTS

### Classification of pelvic nerve activity *in vivo* recorded using the LINE device

#### Electrically evoked pelvic nerve activity

**FIG. 3.**
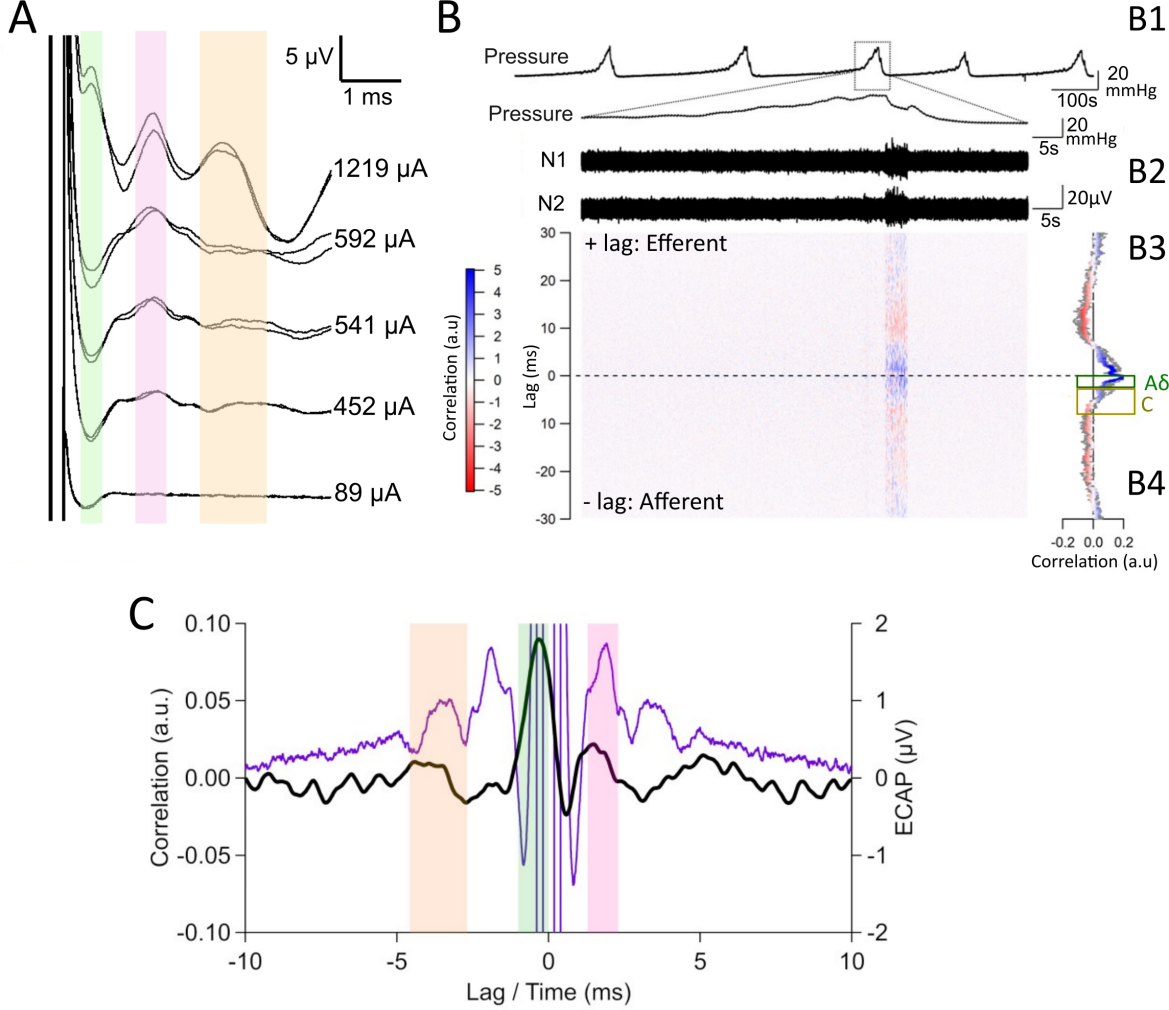
Recordings of electrically and physiologically evoked neural activity in the pelvic nerve. (a) Evoked compound action potential (ECAP) recordings contain three distinct neural populations from the pelvic nerve (green: A-δ, orange: C-fiber, and pink: efferent). (b) From the same animal, pressure traces show five saline-induced voids evoked during continuous cystometry testing. Bipolar recordings of pelvic nerve activity were taken from E1 and E2 (closest to the spine, Rec 1) and E3 and E4 (closest to bladder, Rec 2). The resultant heatmap illustrates the strength of the correlation, with red indicating a strong negative correlation and blue a strong positive correlation at the corresponding lag. The average cross correlation from an individual void (colored trace) and all voids (gray traces) show slow afferent C-fiber (indicated by orange box) and fast afferent Aδ (indicated by green box) activity. (c) The neural activity of an individual void (black trace) is overlayed with a suprathreshold ECAP (purple trace) taken from the same animal to demonstrate similar timing of waveform peaks (afferent A-δ: 1 vs 1.1 ms; C-fiber: 5.7 vs 5.7 ms; and efferent: 1.5 vs 2 ms). The ECAP response is reflected around zero for display purposes. Green shading indicates Aδ activity, orange shading indicates C-fiber activity, and pink shading indicates efferent activity.

Stimulating the pelvic nerve with E1 and E2 [closest to the spine, Fig. 1(b)] and recording from E3 and E4 (closest to the bladder, S12R34 in Table I) evoked up to three distinct waveforms, categorized by latency, similar to that reported previously (Payne *et al.*, 2020). A representative example in Fig. 3(a) shows waveforms evoked during suprathreshold stimulation had a positive peak at 1.1 ms (P1 indicated by green shading, threshold = 128 *μ*A), at 2.0 ms (P2 indicated by pink shading, threshold = 128 *μ*A), and at 3.7 ms (P3 indicated by orange shading, threshold = 263 *μ*A). Data from our analysis of evoked waveforms recorded from n = 12 rats are summarized in Table I.

No difference in stimulation threshold measured with the two electrode configurations S12R34 and S34R12 (Table I) was detected by a mixed-effects model (main effect of electrode configuration: *F*_[1,11]_ = 1.94; interaction of configuration × neural population: *F*_[1.2, 3.0]_ = 1.01, *P* = 0.409), but a main effect of neural population (*F*_[1.7, 19.0]_ = 5.01, *P* = 0.021) was attributed to an increase in thresholds between P1 and P2 (Sidak's *post ho*c test: S12R34, *P* = 0.054 and S34R12, electrode *P* = 0.009).

A comparison of recording efficiency (Table I) showed evoked waveforms recorded using electrode configuration S12R34 show P1 and P2 responses were reliably recorded in most rats (n = 12), while P3 was only recorded in 42% of rats suggesting the slower P3 activity was more difficult to record (Table I). Waveforms recorded using electrode configuration S34R12 were more variable with P1 responses recorded in only 67% of rats, while P2 was recorded in 100% of rats and P3 in 58% of rats (Table I).

Similar latencies of P1, P2, and P3 waveforms were recorded by electrode configurations S12R34 and S34R12 (Table I). When the center-to-center distance of 3.25 mm between electrode pairs E1–E2 and E3–E4 was used to estimate conduction velocities, values for P1 and P2/P3 were in the range reported for Aδ- and C-fiber afferents, respectively (Table I).

#### Classification of physiologically evoked activity using cross-correlation analysis

**FIG. 4.**
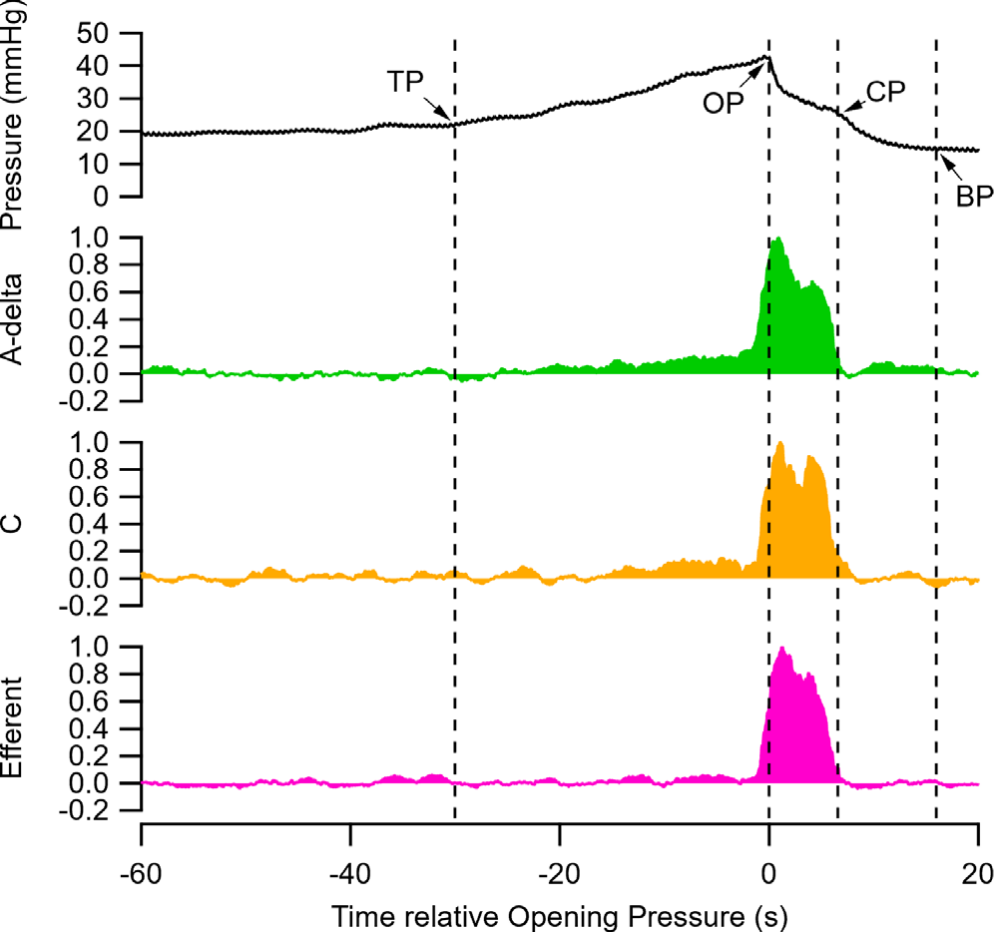
Neural activity extracted using the cross-correlation analysis during saline-induced cystometry. Average (n = 5) pressure change recorded during saline-induced voiding, with arrows indicating threshold pressure (TP), opening pressure (OP), closing pressure (CP), and baseline pressure (BP). Average sliding RMS of the neural response shows corresponding relative neural activity of afferent Aδ fibers (green trace, −1 ms lag), C-fibers (orange trace, −3.55 ms lag) and efferent fibers (pink trace, +1.5 ms). Data show pressure (mm Hg) and neural RMS (arbitrary units).

We next investigated if our cross-correlation analysis methods could extract different classes of physiologically evoked pelvic neural activity recorded when cystometry was used to induce micturition [Fig. 3(b)]. Bladder pressure was recorded during slow filling via a bladder catheter, and five reproducible voids were used to establish baselines [Fig. 3(b-1)]. Data from cross-correlation analysis of bipolar recordings [Rec 1 and Rec 2, Fig. 3(b-2)] generated during a single, representative void were displayed as a heat map [Fig. 3(b-3)]—with strong positive correlations indicated by brighter blue colors and strong negative correlations indicated by brighter red colors. The average cross correlation of the entire recording segment derived from these data for a single void was also plotted [Fig. 3(b-4), colored trace] and compared to the cross correlations from all five voids [Fig. 3(b-4), gray traces]. The average cross correlation of the individual void exhibited a peak at lag −1 ms, indicating this neural population was afferent and had an approximate conduction velocity of 3.25 m/s, which is in the range for Aδ activity in the rat pelvic nerve [Fig. 3(b-4) indicated by a green box]. A second peak at lag −3.56 ms was afferent with an approximate conduction velocity of 0.91 m/s in the range of C-fiber activity [Fig. 3(b-4), indicated by an orange box] (Vera and Nadelhaft, 1990; Sengupta and Gebhart, 1994; and Shea *et al.*, 2000). A peak with a positive lag (+1.5 ms) indicated it was efferent with an approximate conduction velocity of 2.17 m/s.

To validate the extracted neural data in Fig. 3(b), the neural activity of an individual void [Fig. 3(c), black trace] was overlayed with a suprathreshold representative electrically evoked neural recording [Fig. 3(c), purple trace] taken from the same animal. The electrically evoked compound action potential (ECAP) waveform peaks align with peaks in the cross-correlation analysis (lags), with green shading indicating fast afferent, i.e., Aδ activity (ECAP latency: 1.1 ms, cross-correlation lag: −1.0 ms), orange shading that of slow afferent, i.e., C-fiber activity (ECAP latency: 3.7 ms, cross-correlation lag: −3.7 ms), and pink shading efferent [ECAP latency: 2.0 ms, cross-correlation lag: +1.5 ms, Fig. 3(c)]. The ECAP in Fig. 3(c) is reflected around zero for display purpose.

### Selective extraction of simultaneous patterns of neural activity during voiding

Exemplar neural responses extracted from the heatmaps from five saline induced voids were averaged to show corresponding relative neural activity (Fig. 4). Minimal afferent or efferent neural activity was detected during filling, i.e., before threshold pressure (TP) was reached. When threshold pressure, which is the pressure reached at the beginning of the voiding contraction, is reached there was a small linear increase in Aδ activity (−1 ms lag, approximate conduction velocity of 3.25 m/s, Fig. 4: green trace) that coincides with the increase in bladder pressure. Maximum Aδ activity was detected during voiding, i.e., between opening pressure (OP) and closing pressure (CP), in which urine is expelled in pulsatile bursts in rats. Following expulsion of urine Aδ activity continues during relaxation of the bladder back to baseline pressure (BP), after which Aδ activity abruptly ceases. C-fiber activity (−3.55 ms lag, approximate conduction velocity of 0.91 m/s, Fig. 4, orange trace) was minimal prior to TP and only began to increase ∼tens after Aδ activity began. C-fiber activity also exhibited a more rapid decrease than Aδ activity following CP, with little evidence of activity between CP and BP. The majority of efferent activity (+1.5 ms lag, approximate conduction velocity of 2.17 m/s, pink trace) was evident just prior to OP and during the voiding phase (OP to CP), activity ceasing abruptly during the relaxation phase of voiding (OP to BP).

### Specificity of neural recording and analysis technique

We next determined if our cross-correlation analysis could detect changes in neural activity following experimental pharmacological or surgical interventions that increased or inhibited activity in different subpopulations of fibers in the pelvic nerve [Fig. 1(a), summarized in Table II]. Specifically, afferent activity was increased by continual intravesical infusion of capsaicin (0.3 *μ*M) or PGE2 (100 *μ*M) during cystometry but decreased or silenced by application of pharmacological drugs (TTX, lidocaine) or a cut injury to the accessory nerve [distal lesion, Fig. 1(a), Table II]. Efferent activity was decreased or silenced following intervention to the pelvic nerve [proximal lesion, Fig. 1(a), Table II].

**TABLE II.** Summary of expected changes in neural fiber populations in the pelvic nerve following pharmacological and mechanical intervention.^a^

Fiber type	Class	Capsaicin (0.3 *μ*M)	TTX (3 *μ*M)	PGE2 (100 *μ*M)	Distal nerve cut	Proximal nerve cut
Afferent	Aδ	↑^b^ (Weak)	¯ (Block)	↑ (Direct/indirect)	¯ (Block)	⋯
	C	↑ (Strong)	⋯	↑ (Direct/indirect)	¯ (Block)	⋯
Efferent	B/C	↑ (Indirect)	¯ (Block)	⋯	⋯	⋯ (block)

^a^ Summary of data from Maggi *et al.*, 1984; Mallory *et al.*, 1989; Vera and Nadelhaft, 1990; Sengupta and Gebhart, 1994; Le Feber *et al.*, 1997; van Asselt *et al.*, 1999; Shea *et al.*, 2000; Yoshimura *et al.*, 2003; Le Feber *et al.*, 2004; Kuga *et al.*, 2016; and Kuga *et al.*, 2017.

^b^ Symbols represent changes in activity compared to baseline (pre-intervention levels): – minimal or no change in activity; ↑ increase in activity; ↓ decrease in activity.

Prior to a pharmacological or mechanical intervention, stable baseline, i.e., control voiding was established (Andersson *et al.*, 2011; Fraser *et al.*, 2020) and summarized in supplementary material Table 1, (n = 12 total). These control (baseline) urodynamic parameters, including intervoid interval (IVI), threshold pressure (TP), opening pressure (OP), closing pressure (CP), and baseline pressure (BP) were measured during urethane anesthesia. Differences in urodynamic parameters following an intervention were tested using an RM one-way ANOVA (outcomes summarized in supplementary material Table 1).

#### Increasing afferent activity

**FIG. 5.**
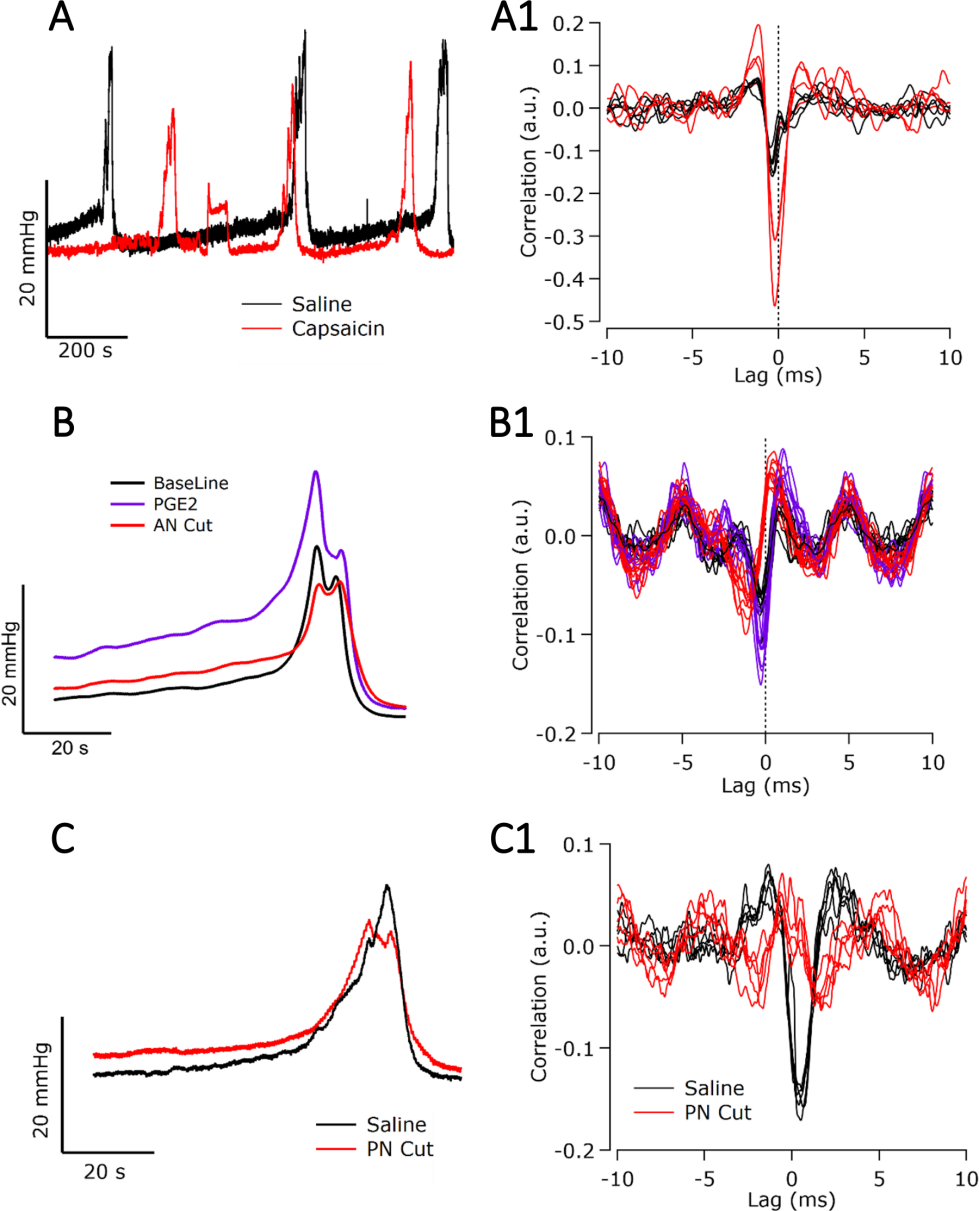
Changes in neural activity following pharmacological or mechanical intervention that increased or inhibited neural activity in the pelvic nerve. (a) Representative pressure traces during saline (control, black trace) and capsaicin-induced voiding (red trace). (a-1) Corresponding correlation of physiologically evoked neural activity during saline infusion (black trace) has a peak at −0.2 ms lag (16.3 m/s) and is indicative of fast afferent Aδ activity. The strength of the −0.2 ms peak correlation increased during intravesical infusion of capsaicin, indicating an increase in relative Aδ activity (red trace). (b) Averaged pressure traces from three consecutive void events during intravesical infusion with saline (control, black trace) and PGE2 infusion (purple trace). In the same animal, the accessory nerve was later cut (AN, red trace). (b-1) Corresponding correlation of neural activity during saline intravesical infusion produces a peak at −0.3 ms lag (10.8 m/s), indicating fast afferent Aδ activity. During PGE2 infusion the strength of the −0.3 ms lag correlation increases, indicating a relative increase in Aδ activity. Following an accessory nerve cut, relative Aδ activity is reduced, but not abolished. (c) Averaged pressure traces from three consecutive void events during intravesical infusion with saline (control, black trace) and after proximal pelvic nerve cut (red trace). (c-1) Corresponding correlation of an efferent response at +1.5 ms lag (2.16 m/s) during saline infusion (black trace) was abolished after the pelvic nerve was cut (PN cut, red trace) proximal to the recording site.

Infusion of intravesical capsaicin directly activates bladder nociceptor afferents, which are mostly C-fibers but may also include a small subpopulation of Aδ fibers (Shea *et al.*, 2000; Aizawa *et al.*, 2011). A representative example of a pressure trace and neural response averaged from three voids recorded before and after infusion of intravesical capsaicin (0.3 *μ*M) are shown in Fig. 5(a). In the example shown in Fig. 5(a-1), there was a significant increase (Student t-test, *p* = 0.039, n = 5 voids) in the peak correlation of fast afferent fibers (−0.2 ms lag, 16.3 m/s conduction velocity) during capsaicin infusion [Fig. 5(a-1), red trace] compared to saline infusion [Fig. 5(a-1), black trace]. As described in Table III, rats displayed an increase in activity of fast afferent activity (lags tracked at −0.4 and −0.25 ms) during capsaicin infusion, with an overall increase in activity (compared to saline control) of +185 ± 26.4% (n = 3 rats). These data support that our cross-correlation analysis methods were reliable in extracting capsaicin-sensitive Aδ activity (n = 3 rats total). However, no distinct correlation peak corresponding to the slower C-fibers was seen.

**TABLE III.** Data summary of changes in neural activity following interventions that increase, decrease, or eliminate responses during continuous cystometry testing. Data show number of subjects, number of voids analyzed from control (saline), or intervention voids and how many neural responses were extracted from these voids. The lag range of neural responses (ms) is reported. The change in the peak correlation between control and intervention is displayed as a percentage mean ± S.E.M, with a + percentage indicating an increase (↑) and negative percentage indicating (↓).

Parameter (units)	Capsaicin	PGE2	Accessory nerve intervention	Pelvic nerve intervention
Subjects	3	2	5	3
No. control voids	13	9	20	13
No. control neural responses	11	7	18	13
No. intervention voids	15	9	21	10
No. intervention neural responses	14	8	2	2
Lag range (ms)	−0.2 to −0.4	−0.34 to −0.53	−0.88 to −1.1	+1.45 to 2.30
Correlation change from control (%)	+185 ± 26.4	+40 ± 24.0	−76 ± 18.1	−90 ± 10
	↑	↑	↓	↓

PGE2 increases the activity of a subpopulation of Aδ fibers usually silent during voiding in normal physiological conditions as well as C-fibers [Tables II and III and Fig. 5(b)] (Kuga *et al.*, 2016; Kuga *et al.*, 2017). In the example shown in Fig. 5(b-1), PGE2 significantly increased (Student t-test, *p* = 0.031, n = 5 voids), the peak correlation of fast afferent neural activity (−0.3 ms lag, approximate conduction velocity 10.8 m/s) compared to saline control [Fig. 5(b-1), black trace]. In both rats, we detected an increase in +40% ± 24% in the peak correlation of extracted afferent activity (n = 2 rats, Table III).

#### Inhibiting afferent activity

We first determined the effect of cutting the accessory nerves between the bladder and the recording site [Fig. 1(a)], which was performed in five rats. This blocks transmission of all afferent signals in the pelvic nerve that are received from the bladder and other pelvic viscera via the accessory nerves. As this nerve transection is unilateral, urodynamic activity during cystometry is compromised but is otherwise maintained by the intact pelvic nerve on the contralateral side. In the example shown in Fig. 5(b-1), an accessory nerve cut significantly reduced (Student t-test, *p* = 0.029, n = 5 voids) the peak correlation of fast afferent neural activity (−0.3 ms lag red trace), which had previously been infused with intravesical PGE2 (purple trace, Table III). In three out of four rats, including animals, fast afferent responses were abolished following accessory nerve cut. In the remaining one of four rats, the correlation of the afferent response decreased by –73%, compared to control (saline) correlation values.

Application of TTX to the accessory nerve (n = 1), which selectively blocks voltage-gated sodium channels and silences Aδ-fiber activity (Kuga *et al.*, 2016), resulted in a small −7% decrease in the correlation of A-δ activity (16.3 m/s), compared to control correlation values.

#### Inhibiting efferent activity

We next examined the effects of blocking efferent activity in the pelvic nerve proximal to the recording site [n = 4, Fig. 5(c)]. To do this, we abolished all preganglionic efferent activity from the spinal cord by cutting the pelvic nerve proximal to the electrode. In the example shown in Fig. 5(c-1), a pelvic nerve cut significantly reduced (Student t-test, *p* < 0.001, n = 5 voids), the peak correlation of efferent neural activity (+1.5 ms lag, approximate conduction velocity: 2.17 m/s), compared to control (saline) correlation values. Similar reductions/abolition of efferent responses were observed in rats after lidocaine (n = 1) or TTX (n = 1) was applied to the proximal pelvic nerve to block efferent action potentials. As described in Table III, the correlation of efferent responses was abolished in two of three rats following pelvic nerve intervention. In the remaining one of three rats, the correlation of efferent responses decreased by 70% compared to control correlation values.

### Correlation of integrated neural activity with bladder pressure

**FIG. 6.**
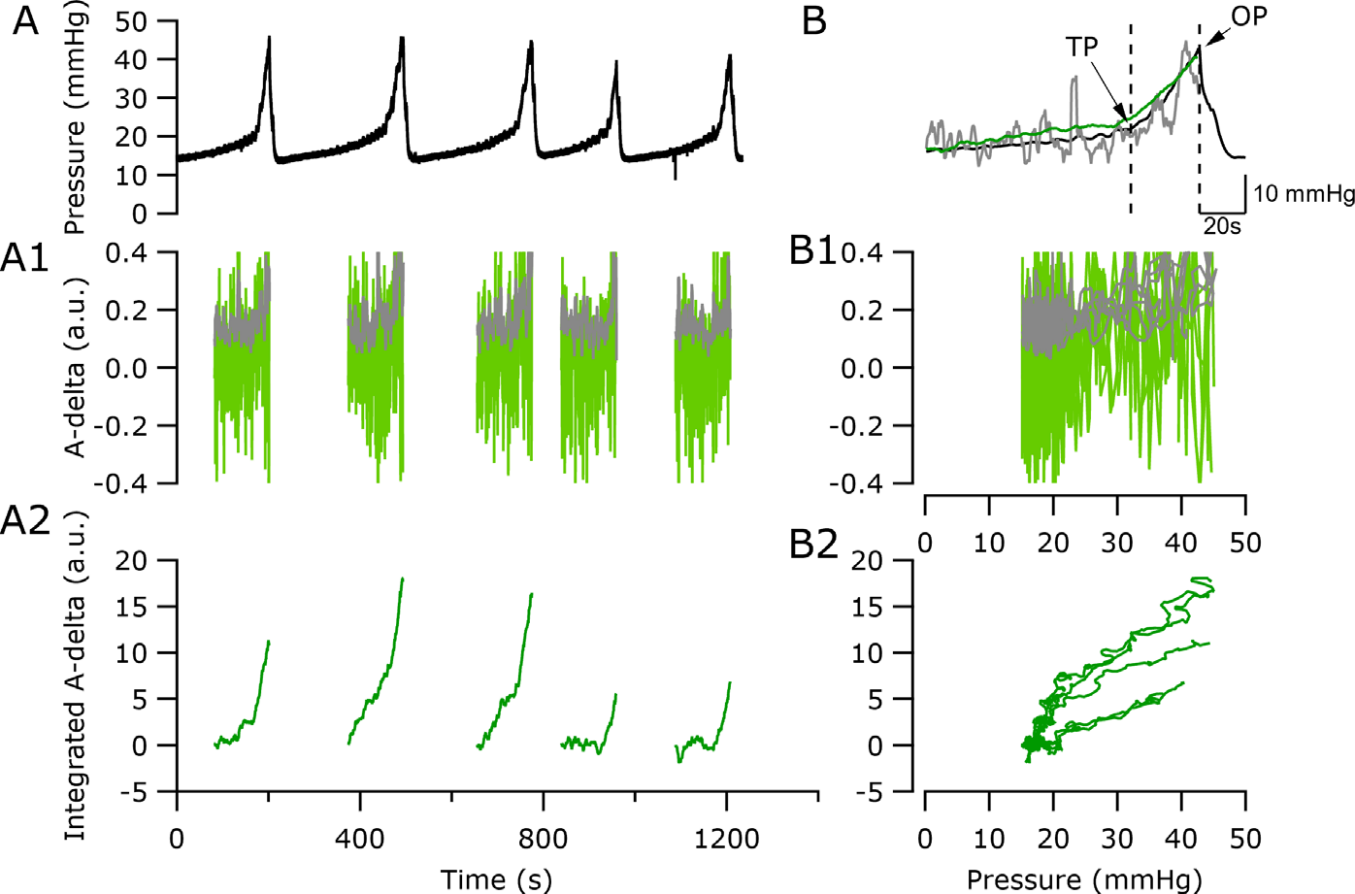
Correlation of extracted afferent activity, RMS of extracted afferent activity, and integrated afferent activity with saline- or capsaicin-induced voiding. (a) Reproducible pressure traces were recorded over time during saline-induced voiding. (a-1) Corresponding extracted neural activity at lag −0.3 ms (fast Aδ afferent at 10.8 m/s, indicated by the green trace) and RMS of neural activity (indicated by the gray trace), were assessed over time for each individual voiding event. (a-2) Extracted Aδ afferent activity was integrated and assessed over time for each individual void. (b) The pressure changes from five saline-induced voids were average (black trace). Threshold pressure (TP) and opening pressure (OP) urodynamic events were overlaid with averaged RMS neural activity (n = 5, gray trace) and averaged integrated Aδ activity (−0.3 ms lag, n = 5, green trace). (b-1) Individual extracted Aδ activity (−0.3 ms lag, green traces) and RMS of neural activity (gray traces) are overlaid and correlated with pressure during the voiding event. (b-2) Integrated Aδ activity is correlated with pressure from individual voids.

A key outcome of this study was to develop a real-time neural based biomarker that accurately informs on the physiological state of the bladder as a first step in the development of closed-loop control technology. Our LINE recording device and cross-correlation analysis was used to extract neural activity during saline infusion during cystometry testing [Fig. 6(a)]. In Fig. 6, a representative example shows changes in extracted afferent activity and 2-s (rectangular window, with 0.5-s overlap) sliding RMS of the extracted afferent activity [Fig. 6(a-1)] and a short-term (from 120 s prior to OP to OP) integration of the extracted afferent activity [Fig. 6(a-2)] over time during five saline-induced voids. Neural analysis was limited to the late filling stage (from 120 s prior to OP to OP) to focus analysis on the period during which neural activity is likely to be most informative about bladder state. There was a weak correlation between extracted Aδ (−0.3 ms lag, 10.8 m/s) afferent activity and void pressure bladder [*p <* 0.05, R^2^ = 0.234, Fig. 6(b-1)]. Similarly, there was a weak correlation between the RMS of the extracted afferent activity and pressure during the five voids [Pearson's correlation, *p* < 0.05: R^2^ = 0.488, Fig. 6(b-1)]. In contrast, integration of the extracted afferent activity was strongly correlated with bladder pressure (*p* < 0.05) with an R^2^ = 0.77 [Fig. 6(b-2)].

Analysis from three consecutive voids during saline-induced cystometry of n = 3 rats (urodynamic data indicated in supplementary material Table 1 in the “capsaicin” column) showed integrated Aδ afferent activity significantly correlated with void pressures (*p* < 0.05), which equated to R^2^ = 0.77 (−0.3 ms lag, Rat 1), R^2^ = 0.66 (−0.4 ms lag, Rat 2), and R^2^ = 0.56 (−0.3 ms lag, Rat 3). This gave an average R^2^ of 0.66 ± 0.06 (n = 3 total). The strength of the correlation of integrated Aδ afferent activity with void pressure was assessed following intravesical infusion with capsaicin, which is associated with increases in Aδ (and C-) fiber activity (Shea *et al.*, 2000). The correlation of integrated Aδ afferent activity with void pressures remained similar in two of three rats (Rat 1, saline R^2^ = 0.77 vs capsaicin R^2^ = 0.66; Rat 2: saline R^2^ =0.66 vs capsaicin R^2^ = 0.78). The correlation in Rat 3 was abolished by the presence of capsaicin (saline R^2^ = 0.56 vs capsaicin R^2^ = 0.33).

## DISCUSSION

Autonomic nerves projecting to visceral organs are a major therapeutic target for closed-loop devices (Rijnbeek *et al.*, 2018). There has been a surge in peripheral nerve interface research powered by several government and commercial funding agencies, which have a common goal of developing new tissue interfaces, electronics and algorithms to support stable, safe neural interface performance for 12 months (Waltz, 2016; Naufel *et al.*, 2020). However, despite such efforts, very few devices that interface with the autonomic nervous system have reached the clinic and progress has stalled. A major challenge is the anatomical constraints of these small, thin, and fragile autonomic nerves that render the use of many peripheral nerve interface arrays (i.e., USEA designed for large somatic nerves) as unsafe and clinically unviable. Here, we address the gap in the peripheral neural interface field by demonstrating that our minimally invasive extraneural four-electrode Longitudinal Interface Nerve Electrode (LINE) array in combination with cross-correlation analysis can extract and decode physiologically evoked neural activity in the pelvic nerve of the rat. This signal processing technology reliably decoded fast and slow afferent and efferent neural activity, while extracted integrated Aδ afferent activity correlated with bladder pressure during a void event. Taken together, the LINE and cross-correlation analysis is a new recording and signal processing method that could have application in closed-loop control of disease.

Recording of electrically evoked compound action potentials (ECAPs) is a long-standing method of recording electrically evoked neural activity in humans as a way of verifying stimulation is appropriately activating neural pathways (van Dijk *et al.*, 2007). Our own efforts show stable long-term recording of electrically evoked activity in the vagus nerve (Payne *et al.*, 2018, 2019, 2022) and pelvic nerve (Payne *et al.*, 2020; Eiber *et al.*, 2021) of awake, freely moving animals for up to three months. In this study, up to three waveforms were recorded similar to that reported previously (Payne *et al.*, 2020). The latency of recorded waveforms can be used to estimate the approximate conduction velocity of these three neural populations, which were consistent with that of myelinated Aδ (1.6–21 m/s) and unmyelinated C-fibers (0.5–1.6 m/s) (Shea *et al.*, 2000). Although conduction velocity can be used to identify fiber class from ECAP recordings, the orthodromic direction of the propagation, i.e., fiber type (afferent or efferent) cannot be distinguished, which is an essential component required to allow automatically adaptive patient specific therapy.

In this study, we demonstrated our LINE recording and cross-correlation analysis technique extracted multiple classes and types of fibers, that being fast afferent, slow afferent, and efferent activity during saline-induced voiding (Fig. 3). The estimated conduction velocities, based on the latency of the peak in the cross correlation and electrode spacing, of the fast (∼3.25 m/s) and slow afferents (∼0.91 m/s) C-fibers measured by cross-correlation analysis was indicative of Aδ- and C-fibers, respectively, and is consistent with measurements of conduction velocity made from bladder afferents by electrophysiological single-unit recording. Most of the efferent axons in the pelvic nerves are projections of parasympathetic autonomic preganglionic neurons in the lumbosacral spinal cord. Evidence from electron microscopy suggests most of these preganglionic axons are unmyelinated but around 20% are myelinated, whereas all of the sympathetic autonomic postganglionic axons originating from paravertebral chain ganglia are unmyelinated (Hulsebosch and Coggeshall, 1982). Myelinated parasympathetic axons in visceral nerves or rat and other species have been identified as B-fibers and are reported to have maximal conduction velocity of 5 m/s (Mallory *et al.*, 1989). In this study, the estimated conduction velocity of pelvic nerve efferents was 2.15 m/s, but we could not verify if these recordings were mostly from myelinated efferent fibers.

Studies using invasive multiunit recordings from postganglionic bladder nerves to measure neural activity evoked during saline-induced voiding provide a basis for comparison to assess the accuracy and validity of our extracted neural recordings (Le Feber *et al.*, 1997, 2004; Zvara *et al.*, 2010; and Choudhary *et al.*, 2015). Specifically, our observed pattern of no detectable activity during the filling phase, a modest build in Aδ and C-fiber activity after threshold pressure is reached and active contraction of the bladder begins, with a peak in activity during the expulsion of urine [i.e., between the opening and closing pressure of the cystometry trace, Fig. 2(b-1)] corresponded well to those reported in the literature using invasive techniques. During the post-void relaxation phase (i.e., following the closing pressure), we and others report a complete cessation of activity after the closing pressure event. However, our recording of efferent activity was seen only during phase II, while previous studies, using far more invasive recording techniques, show a clear linear increase in post-ganglionic efferent activity during the contraction of the bladder (between threshold pressure and opening pressure) as well as during phase II (Choudhary *et al.*, 2015). Despite this, we have demonstrated a recording and analysis technique that distinguished between fiber type and class, which has not been achieved previously without cutting the nerve to isolate neural activity (Le Feber *et al.*, 1997; Zvara *et al.*, 2010).

An extraneural cuff array (PDMS Sling μCuff, CorTec) has also been used to record and extract activity from the surface of the whole mouse vagus nerve (Steinberg *et al.*, 2016). Unique, distinctive neural patterns were recorded in response to injections with several different inflammatory-inducing immune stimuli (Steinberg *et al.*, 2016) and in a model of sepsis (Zanos *et al.*, 2018). More recently, recorded vagal activity correlated with insulin-induced acute hypoglycemia (Masi *et al.*, 2019). While promising, the technique does not discriminate between fiber type, i.e., sensory and motor, and the conclusion that neural activity was afferent was only made after a reduction in signal following cutting the afferent input (distal cut) (Steinberg *et al.*, 2016). Finally, the class (A-, B-, and C-) of fibers was not determined.

The velocity selective recording (VSR) approach has been shown to differentiate between both fiber type and class in the ulnar nerve in pigs (Andreis *et al.*, 2021; Metcalfe *et al.*, 2021). The technique described in this work shares some similarities with VSR (namely, the use of timing cues between electrodes) to determine fiber type and direction of activity (i.e., fiber class) in a large somatic (Andreis *et al.*, 2021) and autonomic nerve (Metcalfe *et al.*, 2018), albeit with the requirement that VSR utilize a large number (14 and 10, respectively) of electrodes. However, VSR and the method described in this work aim to extract neural activity in fundamentally different ways. VSR uses multiple electrodes to enhance the SNR of the signal (with SNR scaling approximately with the square-root of the number of electrodes) and then utilizes threshold crossing analysis of spiking activity. The ability of VSR to sufficient improve SNR to work on the smaller, slower, and more temporally dispersed activity typical of that occurring in small peripheral visceral nerves is yet to be demonstrated. The technique described here utilizes two pairs of electrodes and extracts a population measure of neural activity. Our data support our smaller four-electrode LINE array is effective at extracting physiologically evoked Aδ afferent and efferent activity with small-to-negative SNRs from a small, fragile visceral nerve. The ability to extract C-fiber afferent activity was less robust but was occasionally possible. The ability to extract neural activity with a negative SNR from a visceral nerve represents a significant advance in the neuromodulation field.

Correlation of bladder pressure with extracted integrated neural recordings is a significant first step in developing algorithms that can predict a bladder event. For example, prediction of the threshold pressure is particularly valuable for developing a closed-loop system for bladder control as this micturition event represents the moment in which neural reflexes are engaged to result in voiding of urine. Here, we demonstrate using our minimally invasive LINE and cross-correlation analysis that integrated Aδ (−1 ms lag) afferent activity correlated with void pressure with an R^2^ of 0.66 ± 0.06. The use of integrated Aδ activity resulted in stronger correlations than using extracted afferent activity or the RMS of the extracted afferent activity. The current study is the first to correlate afferent activity with rapid fluctuating changes in bladder pressure during a saline-induced void using an extracellular minimally invasive electrode array. Although a previous study recorded bladder afferent activity during cystometry-induced voiding in awake mice, afferent activity was not correlated with pressure (Zvara *et al.*, 2010). Furthermore, studies show the activity of bladder neurons in sacral DRGs of cats correlated with isovolumetric pressure testing in the bladder (Ross *et al.*, 2016; Sperry *et al.*, 2021), and a Kalman filter-based non-linear model was used to predict bladder pressure with a high correlation (0.81 ± 0.13) (Ross *et al.*, 2018). While we have provided proof-of-principle evidence supporting the feasibility of decoding bladder pressure using our techniques, development of a robust decoding algorithm suitable for closed-loop control requires testing in a range of clinical relevant conditions. As a first step, we also showed the strength of correlation was not affected following intravesical infusion with capsaicin, which increases activity of mostly capsaicin-sensitive C-fibers and a small subpopulation of Aδ fibers (Table II) (Shea *et al.*, 2000) as a model of bladder pain. Additional work to ensure the decoding is robust to other clinically relevant factors (e.g., movement artifacts, long-term changes in neural interface, bladder sensitization) will also be required to support the use of this technology in patients with urinary dysfunction but are beyond the scope of the current work.

A key advantage of our approach of using LINE is that it does not require surgical manipulation of the nerve, making it especially suitable for small, unmyelinated autonomic nerves (Gonzalez-Gonzalez *et al.*, 2018). We have previously demonstrated that long-term implantation of the LINE device for periods up to 8 weeks was well tolerated, with no immunohistochemical signs of damage to the pelvic nerve or adjacent neural tissue of the major pelvic ganglion (Payne *et al.*, 2020). The minimal damage caused by long-term implantation of the LINE device is in contrast to long-term implantation of penetrating arrays (e.g., USEA, TIME, LIFE) that typically elicit vigorous tissue responses resulting in a loss of signal amplitude over extended implantation periods (George *et al.*, 2019; Wendelken *et al.*, 2017). Extraneural cuff arrays around the cervical vagus nerve (Cortec, Freiburg, Germany) are associated with the inhibition of efferent fiber integrity and retrograde transportation (Somann *et al.*, 2018). Although the flat interface nerve electrode FINE mitigates risks associated with penetrating and cuff electrode, the implantation surgical procedure still involves dissection and handling of the nerve into the cuff chamber (Tyler and Durand, 2003; Dweiri *et al.*, 2016). Our LINE device offers a further reduced risk of damage by placement of the array over the top of the nerve (Payne *et al.*, 2020).

A critical next step for translation of this technology is to scale from rodents to larger animal models more representative of the human size. Device development frequently occurs in rats due to extensive knowledge of bladder neural circuitry and relative ease of experimental use that allows rapid prototyping and verification. However, the small size (200–500 g as adults) of rats requires the second stage of device development to be scaled up into a large animal models, such as the pig (>50 kg) (Dalmose *et al.*, 2002; Kitta *et al.*, 2018). Other similar extraneural cuff devices that have the LINE design have been successfully implanted onto the vagus nerve of rat and sheep for periods up to three months with no observed functional or histopathological damage to the implanted nerve (Payne *et al.*, 2018, 2022). As such, long-term implantation with a LINE array is feasible and could be clinically viable. Future studies should consider assessing the long-term stability of our LINE recording and cross-correlation analysis in awake, freely moving animals as a next step in translating this technology for use in closed-loop control of disease or neural feedback for neuroprosthetic systems.

Our LINE recording and cross-correlation analysis methods to extract neural signaling could be applied to other peripheral (autonomic or somatic) nerves for a range of applications. The penetrating USEA device has been used to provide neural feedback and has led to the development of the neuroprosthetic system called the Life Under Kinetic Evolution or the “LUKE arm” (Naufel *et al.*, 2020). However, although the LUKE arm is an exciting development in the field, progress has stalled over safety concerns for the penetrating USEA device and the declining stability of the neural signal over time (Wendelken *et al.*, 2017; George *et al.*, 2019). A second application of our recording and analysis methods is to provide precise, accurate signals to inform on the physiological state of the end organ during health and disease. Peripheral nerves can be harnessed to both sense and control the activity of their target organs (Schulkin and Sterling, 2019), and an emerging application of monitoring peripheral activity is to provide continuous feedback on disease state. Such quasi real-time, objective feedback can serve as a sensing component to feedback to neuromodulation treatment systems to provide adaptive closed-loop control of disease (Bouton and Czura, 2018). As such, future studies should consider assessing the efficacy of our LINE recording and cross-correlation analysis to extract and decode neural activity in other key peripheral nerves, i.e., vagus nerve and carotid sinus (Cracchiolo *et al.*, 2021) or ulnar and sciatic nerve (Raspopovic *et al.*, 2021) as a next step in translating this technology for use in closed-loop control of disease or neural feedback for neuroprosthetic systems.

## CONCLUSION

We developed a clinically viable recording device (LINE) and cross-correlation analysis that allows selective extraction of multiple physiologically evoked neural populations (i.e., afferent, efferent, fast, and slow) from a small visceral nerve. This development in recording technology could have significant implications for providing sensory feedback on the status of bladder. This feedback could neuromodulation technology to provide appropriately timed stimulation to restore bladder function. Such minimally invasive recording technology could also be used to provide autonomous real-time sensory feedback on disease status, essential for a closed-loop technology.

## METHODS

### Design of the electrode array

The design of the array was the same as that reported previously (Payne *et al.*, 2020; Fallon *et al.*, 2020). In brief, the array consisted of two pairs of platinum (99.95%) electrodes contained within a medical grade silicone. Each electrode had an exposed recessed surface area of 1.8 × 0.2 mm^2^ (0.36 mm^2^). Electrode pairs were 0.75 mm apart (i.e., E1–E2, or E3–E4, center to center), while the distance between electrode pairs (E1–E2 to E3–E4, center to center) was 3.25 mm [Fig. 1(b)]. A Dacron embedded silicon tab surrounded the electrodes to allow the array to be sutured to underlying tissue [Fig. 1(c)]. Insulated platinum/iridium (90/10, 50 *μ*m diameter) wires were welded to each electrode and formed a helical cable that traversed to a percutaneous connector.

### Animals and anesthesia

Male Sprague-Dawley rats (8–11 weeks old, Animal Resource Center, Western Australia) were kept on a 12 h light/dark cycle and allowed *ad libitum* access to standard chow and water. Animals (n = 12) were anesthetized using urethane (1.1–1.3 g/kg subcutaneous, Sigma) and kept hydrated (1 ml/100 g Hartman's Solution) and warm for the duration of procedure. At the conclusion of the non-recovery experiment, rats were euthanized (350 mg/kg Lethabarb).

### Surgical procedures

Implantation of the electrode array onto the pelvic nerve and the catheterization of the bladder is an established procedure reported previously (Fallon *et al.*, 2020; Keast *et al.*, 2020; and Payne *et al.*, 2020). In brief, the abdominal cavity was exposed, and the prostate retracted to expose the left or right pelvic nerve. The electrode array was carefully aligned so the length of the nerve ran perpendicular to all four electrodes and was secured using sutures [7–0 silk, Ethicon, Fig. 1(c)]. Immediately following the implantation of the array, the bladder was catheterized by inserting a suprapubic catheter (polyethylene tubing: 0.61 × 0.28 mm^2^, SteriHealth, Victoria, Australia), with a flared end (made by heating the tubing), into the dome of the bladder lumen and secured with a purse-string suture (sterile monofilament suture II polydioxanone; Ethicon, Somerville, NJ, USA). The catheter tubing and the lead wire of the cable were externalized, and the abdominal wall and skin sutured closed.

### Urodynamic manipulation and assessment using cystometry testing

*Manipulation*: Urodynamic examination (Keast *et al.*, 2020) was initiated 3–4 h after the induction of anesthesia and 1–2 h following catheterization. The bladder catheter was connected to an inline pressure transducer (MLT0670, AD Instruments, NSW, Australia) that connected to a filling pump (HA33, Harvard Apparatus, Holliston, MA, USA). The bladder pressure signal was recorded using a transducer (MLT0670, AD Instruments, NSW, Australia) and signal acquisition equipment (Cerebrus, Blackrock, Utah, USA). Baseline urodynamic function was determined by continual infusion of sterile saline (room temperature) at 9 ml/h for 30–60 min, until reproducible voids were recorded (Andersson *et al.*, 2011). Physiologically evoked pelvic nerve activity during cystometry was manipulated (Table II) by intra-vesical infusion of capsaicin [0.3 *μ*M capsaicin in 0.1% dimethyl sulfoxide (DMSO) from a stock of 3 mM in 100% dimethyl sulfoxide (DMSO), Sigma] or prostaglandin E_2_ [100 *μ*M PGE_2_ in 0.1% dimethyl sulfoxide (DMSO) from a stock of 3 mM in 100%, Sigma], which were infused for 30 min (approximately 4.5 ml). Pelvic nerve activity was also manipulated by blocking neural transmission in either (1) the distal pathway from the array to the bladder by cutting the accessory nerves or (2) the proximal pathway extending [Fig. 1(a)] from the array to the spinal cord, either surgically by crushing the proximal pelvic nerve with forceps or pharmacologically using local application of tetrodotoxin (TTX, 1 *μ*l of 3 *μ*M in 0.01% acetic acid and sterile saline, ab120054, Abcam) to block TTX-sensitive action potentials, or lidocaine hydrochloride monohydrate (1 *μ*l of 2% lidocaine in sterile saline, Aspen Pharmacare Aust Pty Ltd) to block all action potentials [Table II, Fig. 1(a)]. Assessment: cystometry data were analyzed using customized IGOR Pro 9 software (WaveMetrics, Inc. Portland, USA). Voiding contractions induced by continuous flow cystometry were recorded and 5–10 continuous voiding cycles were analyzed to determine standard urodynamic parameters. Urodynamic parameters measured included: intervoid intervals (IVI, min), threshold pressure (TP, mm Hg), opening pressure (OP, mm Hg), closing pressure (CP, mm Hg), and minimum pressure (BP, mm Hg) (Andersson *et al.*, 2011; Fraser *et al.*, 2020) (supplementary material Table 1).

### Recordings of electrically and physiologically evoked neural activity

#### Electrode common-ground impedance testing

Functionality of electrodes *in vivo* was tested by passing biphasic current pulses (100 *μ*s per phase and current of 100 *μ*A) between the electrode of interest and all other implanted electrodes (common ground impedance). The peak voltage at the end of the first phase (V_total_) of the current pulse was used to calculate total impedance (*Z*_total_) using Ohm's law (*Z* = voltage/current) (Payne *et al.*, 2019; Fallon and Payne, 2020).

#### Recording of electrically evoked compound action potentials (ECAPs)

Electrically evoked compound action potentials (ECAPs) with complex waveforms were recorded from the non-stimulating electrode pair during graded electrical stimulation in all rats included in this study (n = 12, Table I). As reported previously (Fallon and Payne, 2020), ECAPs were recorded from two electrode pairs (E1–E2 and E3–E4) to confirm that electrodes were in full contact with and correctly aligned to the pelvic nerve [Fig. 1(c)]. A biphasic pulse (100 *μ*s phase, 50 *μ*s interphase gap) was delivered at 10 Hz at currents from 0 to 1.6 mA using a custom made stimulator (Fallon *et al.*, 2018). Two sets of evoked electrophysiological recordings were recorded using an isolated bio-amplifier (ISO-80, World Precision Instruments, FL, USA) averaged from a total of 100 responses, and the recordings were sampled at a rate of 100 kHz and filtered (high pass: 300 Hz; low pass: 5000 Hz; voltage gain 10^2^). The ECAP threshold was defined as the minimum stimulus intensity producing a response amplitude of at least 0.05 *μ*V (Table I) (Fallon and Payne, 2020).

#### Recording and cross-correlation analysis of physiologically evoked activity

Differential bipolar recordings (E1–E2 and E3–E4) of pelvic nerve activity were amplified and filtered (voltage gain 10^2^; high pass 110 Hz, low pass: 3000 Hz) using an isolated bio-amplifier (ISO-80, World Precision Instruments, FL, USA) and recordings made using a signal processer (Cerebrus, Blackrock, Victoria, Australia). The cross correlations of large segments of neural recordings (typically 30 s) were used to identify peaks corresponding to different neural sub-classes and were displayed and analyzed in customized IGOR Pro 9 software (WaveMetrics, Inc. Portland, USA). Based on the orientation of the electrode array (Fig. 2), peaks in the cross correlation at positive lags correspond to efferent activity, while peaks at negative lag correspond to afferent activity. The specific lag of the peak corresponds to the conduction delay between the recording pairs and therefore can be used to estimate conduction velocity. Shorter segments of recordings were then cross correlated (typical segment length 50 ms, segment overlap 25 ms) to produce a neural heatmap (Fig. 2). Time-varying activities for different neural sub-classes were then extracted from the heatmaps by tracking the cross correlation at the appropriate lag.

In a representative model of the cross-correlation signal processing method, synthetic recordings were generated by combing stereotypical neural signals from efferent (Fig. 2, Eff: pink trace), fast- and slow-afferent (Fig. 2, Aff: green trace), and electromyographic (Fig. 2, EMG: yellow trace) activity and Gaussian noise (Fig. 2, gray trace). Individual stereotypical signals were modeled by single cycles of a 500–600 Hz sinusoid repeating at 5–20 times per second. Population responses were then generated by combing 1000 individual signals of each neural type after applying a random jitter (≤ 100 ms) and appropriate delays [to represent efferent (1 ms), and fast (1 ms)- and slow (4 ms)-afferent] to the precise timing of the sinusoids for individual signals. The synthetic whole nerve data (Fig. 2, Rec, Rec 2) had a low signal-to-noise ratio. However, the resultant cross-correlation analyses between the pairs of recordings can be displayed using heat maps to show (modeled) neural activity or “hot spots” at time lags that correspond to the conduction delay between our recording electrodes. Specifically, a negative lag indicates afferent activity (green circles), a positive lag efferent activity (pink circle), and zero lag far field electromyography (EMG) activity, a component of which is from rapid opening and closing of the external sphincter during the expulsion of urine. The lag can be used to estimate approximate conduction velocity [lag/distance between electrode bipolar pairs (3.25 mm)] and therefore allows classification of the fiber type (fast and slow, dotted vs dashed green circles, respectively). For example, for recordings using our array, a peak with a lag of −1 ms corresponds to afferent activity with an approximate conduction velocity of 3.25 m/s and therefore would correspond to Aδ activity (Shea *et al.*, 2000).

### Statistics

Differences between neural thresholds between different neural populations and electrode configurations were tested using a mixed effects model and Greenhouse–Geisser correction, and a Sidak *post hoc* test. Differences between normally distributed cystometry data were tested using a one-way RM ANOVA with appropriate *post hoc* tests, stated in the results. A Pearson's correlation test was used to correlate integrated neural activity with bladder pressure, and a paired Student t-test to test for differences. A Student t-test was used to test for difference between control and intervention voids described in Fig. 5. Details of each statistical test are stated in the relevant results section. Statistically significant differences were accepted as *P*-values less than 0.05, and GraphPad Prism 4 (GraphPad Software, USA) or Igor Pro 9 (WaveMetrics, Inc., Portland, USA) used for all analysis.

## SUPPLEMENTARY MATERIAL

See the supplementary material for data describing analysis on urodynamic parameters during continuous cystometry testing in urethane-anesthetized male rats. Changes in urodynamic parameters were measured during intravesical infusion of saline (control), capsaicin or PGE2, and pharmacological or mechanical intervention to the accessory nerve or proximal to the pelvic nerve (Andersson *et al.*, 2011; Keast *et al.*, 2020).

## Data Availability

The data that support the findings of this study are available from the corresponding author upon request.
